# A Non-thermal Biocompatible Plasma-Modified Chitosan Scaffold Enhances Osteogenic Differentiation in Bone Marrow Stem Cells

**DOI:** 10.3390/pharmaceutics14020465

**Published:** 2022-02-21

**Authors:** Ihn Han, Juie Nahushkumar Rana, Ji-Hye Kim, Eun Ha Choi, Youngsun Kim

**Affiliations:** 1 Department of Plasma Bio Display, Kwangwoon University, Seoul 01897, Korea; ranajuie06@gmail.com; 2 Plasma Bioscience Research Center, Kwangwoon University, Seoul 01897, Korea; 3 Ellitech Medical Incorporation, Seoul 02584, Korea; hlove3010@naver.com; 4 Department of Obstetrics and Gynecology, Kyung Hee University Medical Center, Seoul 02447, Korea

**Keywords:** non-thermal plasma, biomaterial, chitosan scaffolds, osteogenic differentiation, tissue engineering

## Abstract

Non-thermal biocompatible plasma (NBP) was considered as an efficient tool in tissue engineering to modify the surface of biomaterials. Three-dimensional chitosan scaffolds have been extensively used in different ways because it holds some remarkable properties, including biodegradability and biocompatibility. In this study, we evaluated the osteogenic potential of NBP-treated chitosan scaffolds using two different plasma sources: a dielectric barrier discharge (NBP-DBD) and a soft jet (NBP-J). The surface modification of the scaffold was evaluated using scanning electron microscopy. For osteogenic differentiation of cells, proliferation and differentiation were tested by using bone marrow-derived stem cells (BMSCs). We observed that cell viability using NBP-DBD and NBP-J treated chitosan scaffolds yielded significant improvements in cell viability and differentiation. The results obtained with MTT and live/dead assays showed that NBP-modified scaffold increases cell metabolic by MTT assay and live/dead assay. It also observed that the NBP treatment is more effective at 5 min with DBD and was selected for further investigations. Enhanced osteogenic differentiation was observed using NBP-treated scaffolds, as reflected by increased alkaline phosphatase activity. Our findings showed that NBP is an innovative and beneficial tool for modifying chitosan scaffolds to increase their activity, making them suitable as biocompatible materials and for bone tissue engineering.

## 1. Introduction

Non-thermal biocompatible plasma (NBP) is a powerful tool for the development of innovative approaches in biological and medical fields [1]. NBP can be generated using various ionizing gases. NBP comprises many charged particles, including ions and electrons, reactive oxygen species (ROS), reactive nitrogen species (RNS) (or reactive oxygen/nitrogen species [RONS]), ultraviolet light, as well as electric and magnetic fields [2]. RONS generated by plasma play a significant role in various biological functions and have several applications, including cancer therapy, wound healing, dental care, skin diseases, and sterilization [1,2,3,4,5,6,7,8,9,10]. In tissue engineering, NBP interacts with stem cells to enhance their differentiation [11,12,13,14,15]. In addition, NBP has been used extensively for surface modification of biomaterials [16,17,18].

Surface modification of biocompatible materials is important for their medical implementation. Plasma can be used for surface modification of biocompatible materials in bone tissue engineering, which enhances the activity of these materials. Plasma enhances hydrophilicity, functional groups on the surface, and surface roughness [19]. Surface modifications depend on various parameters of plasma, including the plasma jet type and the gas inlet. Plasma based on argon yields many oxygen-containing polar functional groups [20]. Choi et al. reported that treatment of biocompatible materials with NBP involving air and nitrogen gas enhances hydrophilicity, which results in significantly better osteoblast attachment and proliferation [18]. Lauriault et al. reported that NBP induces micropatterned nitrogen-rich deposition on the surface of a polymer film with enrichment of microphages, chondrocyte adhesion, and proliferation, which can be used for tissue engineering [21]. These results indicated that NBP is an advantageous tool for surface modification of biomaterials used in tissue engineering.

Over the last two decades, three-dimensional (3D) scaffolds have been used in bone tissue engineering due to their specific properties, such as porosity, biodegradability, mechanical durability, and surface chemistry. These scaffolds allow stem cell attachment, proliferation, and differentiation. Scaffolds comprising natural polymers have received particular attention due to their flexibility and ability to degrade into low-molecular weight fragments that can be resorbed inside the body [22]. Scaffolds made of natural polymers have recently become more available and have gained in popularity. The use of these scaffolds is increasing with escalating knowledge regarding their chemical and biological properties, and new biomedical applications for these scaffolds have been investigated. Owing to their useful biological properties, such as biocompatibility, bioactivity, and biodegradability, they are considered to be useful in tissue engineering for the repair or regeneration of several tissue types, including bone, skin, cartilage, liver, nerves, and skeletal muscles. Such scaffolds should have various properties, such as porosity, water retention, biocompatibility, protein adsorption, biomineralization, mechanical strength, and biodegradability, which make them suitable for tissue engineering applications.

Chitosan is a natural polyheterosaccharide copolymer obtained from the alkaline deacetylation of chitin [23]. Chitosan and its derivatives have been verified as excellent and safe candidates for enhancing mucosal and transmucosal deliveries. The biological properties of chitosan, including biocompatibility, non-toxicity, and biodegradability, have been studied in the field of bone regeneration. Enhanced biomaterial features of chitosan, such as mucoadhesion and absorption, correlate with the cationic character of the polymer. Due to its positive charge, chitosan is able to adhere to mucosal surfaces, supporting the interaction of a drug with mucus layers, and allows it to overlay different epithelial surfaces for osteoblast attachment and proliferation. As a bone scaffold material, chitosan is of particular interest because it influences the proliferation and attachment of osteoblast cells as well as the creation of mineralized bone matrix. Functional biomaterial research is based on the advancement and development of scaffolding, which can be useful for renovating or regenerating organs or tissues. Osteogenic differentiation of stem cells is enhanced by chitosan scaffolds in vivo [24,25]. However, the use of chitosan scaffolds in bone tissue engineering requires further advances. In terms of improving chitosan-based biomaterials, surface modifications can help to achieve greater biocompatibility and osteoconductivity. NBP-treatment of 3D chitosan scaffolds could potentially be a new technique to enhance the biological effects, particularly the osteogenic differentiation potential, of these biomaterials. However, the effect of NBP treatment of 3D chitosan scaffolds is largely unknown.

In this study, we examined the effect of NBP treatment of a 3D porous chitosan scaffold on the osteogenic potential of this scaffold using bone marrow-derived stem cells (BMSCs) by evaluating cell viability, metabolic ability, and osteogenic differentiation of BMSCs grown on the NBP-treated scaffolds.

## 2. Materials and Methods

### 2.1. Preparation and Characteristics of the Chitosan Scaffold

The chitosan scaffolds selected for this study were prepared and provided by Dankook University. The method used to prepare a chitosan scaffold has been described in previous reports [26,27]. Chitosan solution was dissolved at 1.5 wt. % in 0.5 *v*/*v*% acetic acid (CHT solution). The CHT solution was mixed with mineral oil/Span80 (14:1% *v*/*v*, MS solution) to enhance its physical properties and dispersion. The blended solution (CHT/MS = 60:10% *v*/*v*) was prepared by stirring at 350 rpm for 1 h after the solutions were added to a mold (with 10-mm inner diameter). Thereafter, it was frozen at −80 °C for 3 h. Next, 0.5 N NaOH was inserted into the mold at 1 °C for 8 h. Thereafter, the scaffold was rinsed in a series of ethanol solutions (100, 80, 60, and 40% *v*/*v*) for 5 h at each stage. In the final step, after washing with distilled water for 48 h, the chitosan scaffold was frozen at −80 °C for 24 h, after which it was lyophilized for 72 h.

### 2.2. Experimental Setup

**Figure 1:**
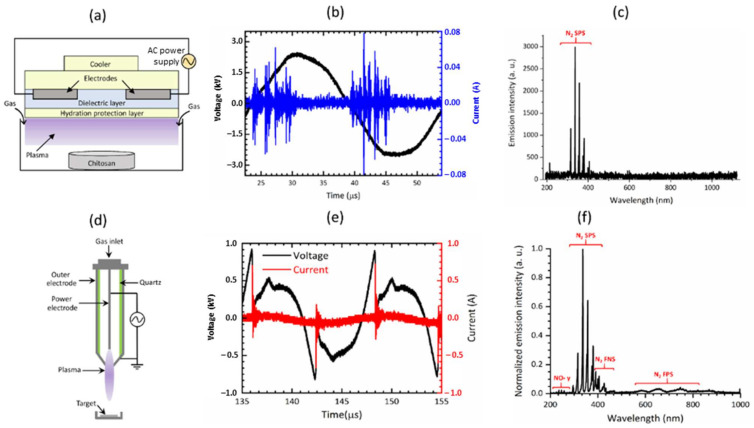
Schematic illustration of the electrical properties of dielectric barrier discharge (DBD) and a soft plasma jet. (**a**) Schematic of DBD, (**b**) current-voltage waveform, and (**c**) optical emission spectrum of DBD. (**d**) Schematic of soft jet, (**e**) current-voltage waveform, and (**f**) optical emission spectrum of the soft plasma jet.

An experimental setup with a dielectric barrier discharge (DBD) and soft plasma jet was used. The configuration and experimental details of DBD are shown schematically in Figure 1a. A 35 mm culture dish was used to place and treat the chitosan scaffolds by using NBP. The mechanism to generate NBP from DBD was described with details in previously reported studies [28,29]. For the generation of NBP, an alternating current power supply was used, and the discharge voltage was determined to be 500 V and discharge current was 13 mA. Nitrogen gas was used as a feeding gas for NBP generation with a flow rate of 1.50 lpm (lpm: liters per minute).

In the case of the soft plasma jet (Figure 1d), the plasma-on time was 25 ms, and the treatment times were 1, 2, 3, 4, and 5 min. Chitosan scaffolds were treated with NBP using a soft plasma jet device at a treatment distance of 2 cm from the ground. The treatment energy at treatment times of 1, 2, 3, 4, and 5 min were 32.4, 64.8, 97.2, 129.6, and 162.0, respectively.

The effects of DBD and soft plasma jet on chitosan scaffolds were observed and compared. The optical emission spectrum (OES) of the plasma produced by the DBD and the soft plasma jet was measured using a spectrometer (model: HR4000, Ocean Optics, Dunedin, FL, USA). The OES intensity of the signals was recorded in terms of the wavelength.

### 2.3. Scanning Electron Microscopy Observation

The characteristics of the control and chitosan scaffolds treated with NBP for 3 min and 5 min with DBD, and for 1, 2, 3, and 4 min with a soft plasma jet were analyzed using scanning electron microscopy (SEM; Hitachi S-3000N, Hitachi, Tokyo, Japan). Three scaffolds of each group (treated by DBD and soft plasma jet) were covered with platinum at 20 mA for 60 s and were then observed using SEM.

### 2.4. Cell Culture with Chitosan Scaffolds

BMSCs were obtained from a normal human osteoblast from the American Type Culture Collection (ATCC, Manassas, VA, USA). The BMSCs were cultured according to previous protocol by I. Han et al. [29]. For the assay, cells were maintained with α-minimal essential medium (α-MEM; HyClone, Logan, UT, USA) and mixed with 10% fetal bovine serum (FBS; Gibco, Waltham, MA, USA) and 1% antibiotics (Gibco, Waltham, MA, USA). Throughout the experiment, the cells were used below passage 8. For DBD, BMSCs were seeded on control and treated scaffolds (3000 μL containing 5 × 10^4^ cells) and were placed in a 6-well plate. For soft jet plasma, cells were seeded in 24-well plates at a density of 1 × 10^5^ cells/mL in 500 μL α-MEM medium. After a 24-h incubation, 900 μL culture media was supplemented into each well, and the culture was maintained at 37 °C, with 95% humidity and 5% of CO_2_. For osteogenic differentiation assays (alkaline phosphatase (ALP) activity and Alizarin red staining (ARS)), the culture medium was replaced with osteogenic-induction medium supplemented with 10% heat-inactivated FBS, 10 mM β-glycerophosphate, and 50 μM L-ascorbic acid 2-phosphate (Sigma-Aldrich, St Louis, MO, USA).

### 2.5. Live/Dead Assay

Cell viability was assessed using a live/dead assay for control scaffolds and those treated with DBD or the soft plasma jet. Initially, the chitosan scaffolds were treated with NBP for 3 and 5 min using DBD. Immediately after the treatment, 5 × 10^4^ cells were cultured on each scaffold and were placed in a 6-well plate. The other cells were cultured at a density of 1 × 10^5^ cells/well in 24 well plates for a soft plasma jet. The treatment times using the soft jet plasma were 1, 2, 3, 4, and 5 min, respectively. The evaluation was carried out after 24 h of incubation using a live/dead cell viability/cytotoxicity assay kit (Molecular Probes, Eugene, OR, USA) according to the manufacturer’s instructions. Before the staining by live/dead kit, cells were washed with phosphate-buffered saline (PBS). Cells were treated with 50 μM calcein, and 2 mM of ethidium homodimer-1 was suspended in 1 mL PBS and kept in room temperature (RT) in dark for 20 min. For image, fluorescence microscope (Nikon, Melville, NY, USA) was used.

### 2.6. Cell Metabolic Activity on Scaffolds

A (3-(4,5-dimethylthiazol-2-yl)-5-(3-carboxymethoxyphemyl)-2-(4-sulfophynyl)-2H-tetrazolium) (MTS) assay was carried out on BMSCs seeded on control and NBP-treated scaffolds (treated for 3 and 5 min with DBD and for 1, 2, 3, and 4 min with a soft plasma jet). MTS was used as a reagent to measure cell metabolic activity. Cells reduce MTS to a soluble formazan compound by metabolically active dehydrogenase enzymes. Absorbance was then measured at a wavelength of 490 nm using a microplate reader (Biotek, Winooski, VT, USA). The results indicated the relative viability of cells on the NBP-treated scaffolds (as % to the control).

### 2.7. Measuring Alkaline Phosphatase Activity

The effect of NBP treatment of scaffolds (by DBD and soft plasma jet) on the ALP activity of BMSCs was assessed using an ALP assay kit (BioVision, Milpitas, FL, USA). BMSCs were seeded at a density of 10^5^ cells/well in culture medium, in 24-well plates, on control and NBP-treated (5 min DBD) chitosan scaffolds. After incubation for 24 h, the cell medium was replaced with osteogenic-induction medium and cells were cultured for 1, 4, and 7 days. At each time point, the ALP activity in the cell supernatant was measured following the assay kit manufacturer’s protocol. A microplate reader (Gen5) was used to measure absorbance at 405 nm and a p-nitrophenol (pNP) standard curve was plotted. ALP activity (U/mL) = A/V/T, where A indicates the amount of pNP generated by the samples (μM), V is the volume of the sample added to the assay well (mL), and T is the reaction time (min).

### 2.8. Determination of Mineralized Matrix

Mineralization of BMSCs on scaffolds modified by NBP generated by DBD was demonstrated by staining with ARS, which selectively binds calcium and produces a bright red color. BMSCs were seeded at a density of 1 × 10^5^ cells/well in culture medium in a 24-well cell culture plate, on control and NBP-treated (5 min DBD) chitosan scaffolds. After 24 h of incubation, the cell medium was changed to osteogenic-induction medium and cells were again incubated for 4 and 7 days. The cells were then washed twice with PBS and were fixed by using 4% paraformaldehyde for 10 min, followed by staining with 2% ARS solution for 1 h at RT. The wells were washed four times with PBS after aspiration of the unincorporated dye. To quantify the calcified matrix, 1 mL of 10% (*v*/*v*) acetic acid was added to each well, after which the plate was incubated at RT for 30 min, with agitation. The absorbance of aliquots (100 μL) of the supernatant were read at 405 nm in a 96-well plate, using a microplate reader (Gen5).

### 2.9. Energy-Dispersive X-ray Spectroscopy Analysis

Chitosan scaffolds were separated into two groups that were treated for 3 min and 5 min by DBD-NBP compared with the control group, which was not treated DBD-NBP. BMSCs were seeded on NBP-treated (3 and 5 min DBD) chitosan scaffolds and control scaffolds. After 24 h, osteogenic induction medium was applied to the cells with scaffold to stimulate the osteogenic differentiation. The mineralization was assessed at 1, 4, and 7 days. For XRD analysis, cells were washed and fixed with Karnovsky’s fixative solution for 2 h. The samples were washed with PBS three times before secondary fixation with 1% osmium tetroxide (OsO4) at RT for 30 min. This was followed by another two washes with PBS. Dehydration in an increasing alcohol gradient (30%, 50%, 70%,80%, 90%, and 100%) was used to remove the water from the scaffolds, after which the samples were dried by using hexamethyldisilane twice, for 10 min each time. The samples (*n* = 3) were coated with platinum at 20 mA for 60 s, and the presence of phosphorus and calcium at the surface was investigated using energy-dispersive X-ray spectroscopy (EDS) (Bruker Nano GmbH, Berlin, Germany).

### 2.10. Statistical Analysis

The data are presented as the mean ± standard deviation of three replicates. Student’s *t*-test was used to compare the control and treated groups. Statistical significance was set at *p* < 0.05. Graphs, calculations, and statistical analyses were performed using Microsoft Excel 365. All the assays included at least three independent experiments for each scaffold group.

## 3. Results

### 3.1. Characteristic of Non-thermal Biocompatible Plasma

Figure 1a shows a schematic representation of DBD. Figure 1b shows the current and voltage waveforms of DBD. The OES of the NBP produced by DBD is shown in Figure 1c. Figure 1d shows the schematic representation of the soft plasma jet. Figure 1e shows the current and voltage waveforms of the soft jet. The OES of the NBP produced by the soft jet is shown in Figure 1f. Discharging N_2_ gas produced NOγ peaks that appeared between the wavelengths of 240 and 280 nm and N_2_ emission lines from the second positive system (SPS) between the wavelengths of 300 and 400 nm, and a hydroxyl radical (OH) at 309 nm. Additionally, the accumulation of nitric oxide (NO) and nitrogen dioxide (NO_2_) increased progressively in a time-dependent manner with NBP discharge when using N_2_ as a flowing gas. In the case of the soft plasma jet, the plasma-on time was 25 ms, and the treatment time was selected as 1, 2, 3, 4, and 5 min.

### 3.2. NBP Treatment of the Chitosan Scaffold Enhances Cell Viability of BMSCs

**Figure 2:**
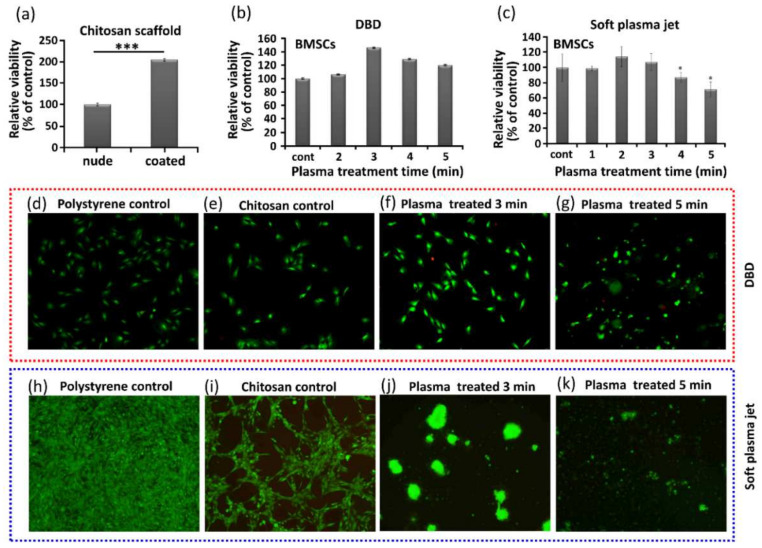
Effect of non-thermal, biocompatible plasma (NBP) on the viability of bone marrow-derived stem cell (BMSC) cultured on chitosan scaffolds. (**a**) Viability of nude and coated cell cultures (i.e., without and with chitosan), (**b**) viability of BMSCs with DBD-based NBP treatment, (**c**) viability of BMSCs with soft plasma jet-based NBP treatment. Live/dead staining to show cell viability of cells cultured on (**d**) polystyrene, as control, (**e**) untreated chitosan scaffold, as control, (**f**) 3-min DBD-based NBP treated chitosan scaffold, (**g**) 5-min DBD-based NBP treated chitosan scaffold, (**h**) polystyrene, as control, (**i**) untreated chitosan, as control, (**j**) 3-min soft plasma jet-based NBP treated chitosan scaffold, and (**k**) 5-min soft plasma jet-based NBP treated chitosan scaffold. Significant differences were indicated as * *p* < 0.05, and *** *p* < 0.0001 by Tukey test after one-way analysis of variance. Data are presented as mean ± SD from three independent experiments.

In tissue engineering, the viability of cells cultured on scaffolds is important for cellular compatibility and appropriateness. We observed that the use of chitosan scaffolds had a significant impact on the viability of BMSCs, as shown in Figure 2, where in Figure 2a, without NBP treatment, the nude and coated labels indicate with and without chitosan, respectively. The viability of the BMSCs in the coated wells was increased significantly. Figure 2b shows the increased viability of BMSCs grown on chitosan treated with NBP generated using DBD. Figure 2c shows the viability of BMSCs grown on chitosan scaffolds treated with NBP generated using a soft plasma jet. The viability of BMSCs also increased after NBP treatment with soft jet. Figure 2d–k shows live/dead staining used to determine viability of cells grown on the control scaffold, and scaffold treated with 3 min and 5 min of NBP generated by DBD and soft plasma jet, respectively. Figure 2d shows the polystyrene control, Figure 2e shows the chitosan control, and Figure 2f,g shows the viability of the BMSCs with NBP treatment generated by DBD at 3 and 5 min, respectively. Most of the live cells showed green fluorescence in the untreated scaffold control group and in the 3-min and 5-min DBD-based NBP-treated scaffolds. The proliferation of BMSCs was enhanced after 3 min, with 3 min of NBP treatment of the chitosan scaffold, while at 5 min, the cells started to migrate inside the chitosan scaffold, as shown in Figure 2f,g, respectively. With the soft plasma jet-based NBP-treated scaffolds, at 3 min (Figure 2j) and 5 min (Figure 2k), there was no significant attachment of cells, as compared to the DBD-treated samples. This is because the scaffold was first directly treated with NBP using a soft plasma jet, and the modified scaffold further affected the cellular behavior. The results obtained indicated that DBD treatment of chitosan resulted in a markedly higher affinity for cells than did the soft plasma jet.

### 3.3. Characterization of Chitosan Scaffold after NBP Treatment

**Figure 3:**
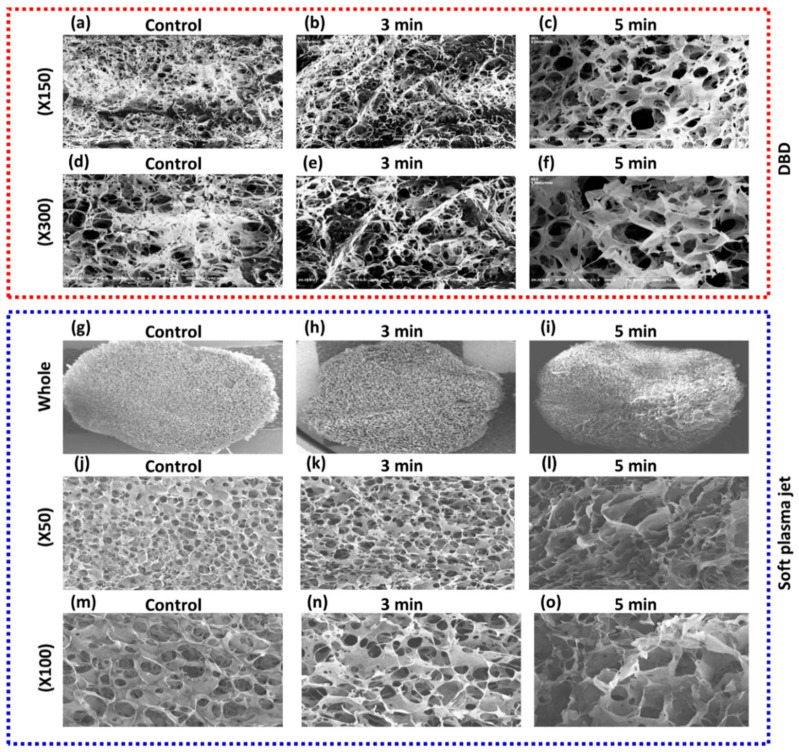
Scanning electron microscopy (SEM) morphology of the 3D chitosan scaffold after non-thermal, biocompatible plasma (NBP) treatment. The chitosan scaffolds were treated with DBD or soft plasma jet NBP for 3 min and 5 min. Non-treated chitosan was used as control. (**a**–**f**) SEM images showing the morphology of control and DBD-based NBP-treated chitosan scaffold. (**g**–**o**) SEM images showing the morphology of control and soft plasma jet-based NBP-treated chitosan scaffold.

SEM micrographs of chitosan scaffolds indicated a highly porous, 3D sponge-like microstructure along with a high degree of interconnection through the scaffold in all directions, as shown in Figure 3. Figure 3a–f shows SEM images of the chitosan scaffolds before and after DBD-based NBP treatment. On the other hand, Figure 3g–o shows the images of the control and soft plasma jet-based NBP-treated chitosan scaffold. The scaffold treated for 5 min with DBD showed a significantly more compact microstructure with a homogeneous pore distribution and thicker barriers between the pores (Figure 3c,f) than that of the scaffold treated with the soft plasma jet. In particular, newly formed surface protrusions were observed on the barriers, which confirmed the increase in surface roughness after 5 min of NBP treatment. However, the 3-min treated scaffold showed heterogeneous pore sizes, as shown in Figure 3b,e. Furthermore, no thermal effect was observed on the scaffolds after 5 min of either type of NBP treatment. This indicated that our DBD and soft plasma jet devices are suitable for modifying chitosan scaffolds without creating thermal effects.

### 3.4. EDS Analysis for Extracellular Mineralization

**Figure 4:**
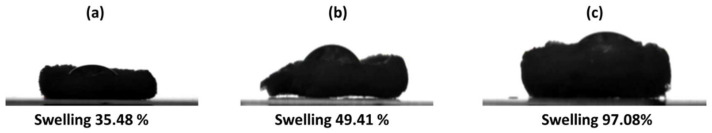
Swelling of chitosan scaffold before and after non-thermal, biocompatible plasma (NBP) treatment. (**a**) Without treatment, the chitosan showed 35.48% swelling, while after NBP treatment, the swelling of the chitosan scaffold increased significantly: (**b**) 49.41% with 3 min treatment and (**c**) 97.08% with 5 min treatment.

EDS analysis was performed to estimate the mineralization of the carbon, nitrogen, and oxygen deposits in the extracellular matrix (ECM) during osteogenic differentiation. Quantitative elemental EDS indicated that the amount of oxygen deposition in the scaffolds increased after NBP treatment (Table 1). However, carbon and nitrogen deposition did not show any changes after NBP treatment. The swelling ratios of the control and treated chitosan scaffolds were also observed before and after the treatment. The swelling ratio was 35.48% in the control, 49.41% in the 3-min NBP-treated, and 97.04% in the 5-min NBP-treated scaffolds, as shown in Figure 4a–c.

### 3.5. Enhanced Osteogenic Differentiation of BMSCs with NBP

**Figure 5:**
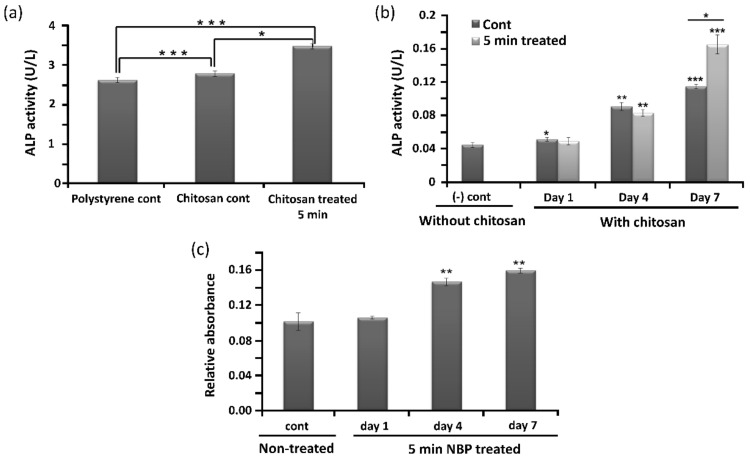
Promotion of osteogenic differentiation of bone marrow-derived stem cells (BMSCs) by non-thermal, biocompatible plasma (NBP)-treatment of the chitosan scaffold. Alkaline phosphatase (ALP) (**a**) activity assay of BMSCs seeded on polystyrene and chitosan control scaffolds, and chitosan scaffolds treated for 5 min with DBD-based NBP. (**b**) ALP activity of control without chitosan and with chitosan (control and 5-min NBP-treated) at 1, 4, and 7 days. (**c**) Detection of mineralization on BMSCs on untreated (control) and 5-min NBP-treated scaffolds at days 1, 4, and 7. Significant differences were indicated as * *p* < 0.05, ** *p* < 0.01, and *** *p* < 0.0001 by Tukey test after one-way analysis of variance. Data are presented as mean ± SD from three independent experiments.

ALP activity is a known osteogenic differentiation marker and is assumed to reflect the degree of differentiation. Figure 5a shows the ALP activity of the polystyrene control, chitosan control, and 5-min DBD-based NBP-treated chitosan. In addition, the ALP activity of BMSCs cultured on the control and 5-min NBP-treated scaffold was measured at 1, 4, and 7 days. The results indicated that the ALP activity of BMSCs cultured on the control and on the NBP-treated scaffolds steadily increased from day 1 to day 7 (Figure 5b). No significant difference was observed between the control and the NBP-treated scaffolds until day 4. However, on day 7 of culture, ALP activity was markedly increased in the 5-min NBP-treated group as compared to the control group. The development of calcified nodules is an important marker for investigating osteoblast maturation. Verification of the mineralization of BMSCs was performed using ARS staining, where the binding of calcium ions in the mineralized ECM forms an ARS-calcium complex via a chelation process. Compared to the control scaffold, 5-min NBP treatment significantly increased the formation of calcified nodules in BMSCs after 4 days under osteogenic-inductive conditions (*p* < 0.001), as shown in Figure 5c.

## 4. Discussion

NBP consists of various particles that include combinations of free radicals, free electrons, ions, and neutral atoms and molecules. RONS in NBP play a significant role in modification of surfaces. These RONS can be observed in NBP using OES. In this study, N_2_ gas was used as the feeding gas. The OES spectrum-excited N_2_ in the second positive system and the NOγ spectrum are shown in Figure 1c,f, respectively. These reactive species react with the chitosan surface groups, resulting in surface modification. Amino and hydroxyl groups are the major groups on the chitosan surface that interact with reactive species and modify the chitosan surface. Amino groups possess a positive charge and can therefore react with negatively charged particles and electrons [22]. NBP possesses a high number of reactive species, most of which belong to ROS or RNS groups, making chitosan a highly reactive polysaccharide. NBP can generate the electrons and RONS and the oxygen and nitrogen groups and polar groups interact with the surface molecules, which increase in surface roughness with sharp pieces of chitosan film [30]. It has been demonstrated that ionized nitrogen and oxygen groups interact on the chitosan surface [31]. That could be effective for the scaffold surface for a given roughness as well as functional modification. In our studies, it is useful for osteogenic differentiation with nitrogen-containing groups produced by NBP and then the treatment of NBP enhanced cell attachment on the wall membrane of the chitosan scaffold. These results were identical to our SEM results, as shown in Figure 3. Chitosan scaffold pore size and thickened pore barriers were changed after exposure to NBP. Morphological changes, such as compactness and thickened interconnectivity, help to increase the affinity of cells for the surface and to increase cell proliferation and viability. It has been reported in several studies that the quality of the surface and microenvironment of chitosan scaffolds, which includes the roughness, matrix elasticity, chemical deposition, and hydrophobicity, can completely influence the behavior cell and direct the osteogenic differentiation of stem cells [32]. The confirmation of cell viability on scaffolds plays a vital role in cellular affinity and appropriateness for the applications in modern tissue engineering [19]. In our study, cell viability increased after being treated for 3 min and 5 min on the scaffold (*p* < 0.05) and for 2 min on the NBP-J treated scaffold. This indicated that NBP treatment rectified the cells that attached and grew on the scaffolds. After attachment and proliferation on the scaffold, the cells differentiated into osteogenic cells by using osteoinductive media. Three major metamorphic phases of cellular proliferation, cell maturation, and mineralization of the ECM occurred during differentiation of BMSCs to the osteogenic lineage, which was accompanied by the upregulation of the osteogenic pathway. The ALP enzyme revealed the initial stages of osteoblast activity and maturation. ALP activity in BMSCs on both scaffolds was upturned in an incubation time-independent manner, and on day 7, ALP activity was higher on NBP-treated scaffold than on the control scaffold in DBD (*p* < 0.001), indicating that treatment with DBD plasma scaffold modification promotes osteogenic differentiation. The last and most important step in osteoblast differentiation is mineralization. Calcium and phosphorus, including mineral matrix, are secreted and deposited in mature osteoblasts. In our study, the beneficial action of NBP-modified chitosan scaffolds on osteoblast ECM mineralization was confirmed by ARS staining and EDS results. BMP-2 also plays an important role in osteogenic differentiation and bone regeneration [33]. The combination treatment of BMP-2 and NBP could stimulate the mineralization to a higher degree than treatment with either single stimulus, suggesting that the combined msethod in vitro could be considered as a basis for the growth of bone in vivo. In the early stages of osteogenic differentiation, ALP is the most common marker [34]. Osterix is an osteogenic-specific transcription factor that is essential for the osteogenic differentiation process and for bone regeneration, contributing to determining osteoblast function and ECM mineralization [35]. The ALP expression level in BMSCs cultured on the NBP-treated scaffold showed that the modification had enhanced the ability to support osteogenic differentiation. Surface microenvironment changes after NBP treatment were effectively examined using SEM to determine the osteogenic potential of the scaffold. It has been observed that the construction of scaffold surfaces can influence cell behavior. The chemical composition of a scaffold could affect its biological properties [22]. In our study, SEM results showed that the scaffolds with NBP modification were associated with surface roughness and exhibited nanoparticle-like structure protrusions. This is in line with our previous study of NBP-treated zirconia, a dental implant material, in which osteogenic potential was improved after being treated with NBP [36]. Engler et al. [37] confirmed that the function of the matrix microenvironment to impact the stem cell differentiation appeared at a late stage, whereas in the initial stages, chemical inductors played a vital task. In this study, we observed that NBP-treated scaffolds had an enhanced ability of supporting the osteoblast phenotype, shown by the higher level of ALP activity. Taken together, NBP is a key tool for fine-tuning the scaffold surface and enhancing its osteogenic potential. In the future, NBP will be a powerful tool for surface modification and could be a candidate for in vivo studies of bone implant engineering.

## 5. Conclusions

The results of this study showed that an NBP-modified scaffold increased cell metabolic activity. The treatment of the chitosan scaffold with DBD for 5 min showed a significant effect. Enhanced osteogenic differentiation of BMSCs was observed when using NBP-treated scaffolds, as indicated by increased ALP activity. The findings of this study suggested that NBP is an innovative and advantageous tool for modifying chitosan scaffolds to increase activity, making these scaffolds suitable as biocompatible materials and bone tissue engineering.

## Figures and Tables

**Table 1:** Chitosan carbon, nitrogen, and oxygen levels before and after 3- and 5-min non-thermal, biocompatible plasma (NBP) treatment.

Element	Control	3-min NBP	5-min NBP
Carbon (wt. %)	24.91	24.65	22.73
Nitrogen (wt. %)	8.20	7.36	7.47
Oxygen (wt. %)	8.94	7.34	10.58

## Data Availability

Data are available on request due to restrictions such as privacy or ethics.
